# Preliminary Results of Initial Testing for Coronavirus (COVID-19) in the Emergency Department

**DOI:** 10.5811/westjem.2020.3.47348

**Published:** 2020-03-27

**Authors:** Vaishal M. Tolia, Theodore C. Chan, Edward M. Castillo

**Affiliations:** University of California, San Diego, Department of Emergency Medicine, San Diego, California

## Abstract

**Introduction:**

On March 10, 2020, the World Health Organization declared a global pandemic due to widespread infection of the novel coronavirus 2019 (COVID-19). We report the preliminary results of a targeted program of COVID-19 infection testing in the ED in the first 10 days of its initiation at our institution.

**Methods:**

We conducted a review of prospectively collected data on all ED patients who had targeted testing for acute COVID-19 infection at two EDs during the initial 10 days of testing (March 10–19, 2020). During this initial period with limited resources, testing was targeted toward high-risk patients per Centers for Disease Control and Prevention guidelines. Data collected from patients who were tested included demographics, clinical characteristics, and test qualifying criteria. We present the data overall and by test results with descriptive statistics.

**Results:**

During the 10-day study period, the combined census of the study EDs was 2157 patient encounters. A total of 283 tests were ordered in the ED. The majority of patients were 18–64 years of age, male, non-Hispanic white, had an Emergency Severity Index score of three, did not have a fever, and were discharged from the ED. A total of 29 (10.2%) tested positive. Symptoms-based criteria most associated with COVID-19 were the most common criteria identified for testing (90.6%). All other criteria were reported in 5.51–43.0% of persons being tested. Having contact with a person under investigation was significantly more common in those who tested positive compared to those who tested negative (63% vs 24.5%, respectively). The majority of patients in both results groups had at least two qualifying criteria for testing (75.2%).

**Conclusion:**

In this review of prospectively collected data on all ED patients who had targeted testing for acute COVID-19 infection at two EDs in the first 10 days of testing, we found that 10.2% of those tested were identified as positive. The continued monitoring of testing and results will help providers understand how COVID-19 is progressing in the community.

## INTRODUCTION

On March 10, 2020, the World Health Organization (WHO) declared a global pandemic due to widespread infection of the novel coronavirus COVID-19 (coronavirus disease 2019) internationally. Due to a number of challenges, including the unpredictable availability of testing materials, testing for the acute infection was sporadic in the early days of the epidemic in the United States. As a result, testing in emergency departments (ED) has been limited and only began to increase following the WHO declaration. We report the preliminary results of a targeted program of COVID-19 infection testing in the ED in the first 10 days of its initiation at our two institutional EDs.

## METHODS

We conducted a review of prospectively collected data on all ED patients who had targeted testing for acute COVID-19 infection at two EDs, located at an urban teaching hospital (ED census approximately 50,000/year), and academic quaternary medical center (ED census approximately 35,000/year) in San Diego, California, within the same healthcare system during the initial 10 days of testing (March 10–19, 2020). During this initial period with limited resources, testing was targeted toward high-risk patients with the following known criteria as per Centers for Disease Control and Prevention (CDC) guidelines: patients presenting with symptoms concerning for COVID-19 infection (fever AND cough or shortness of breath); travel within 14 days to countries with high rates of infection (at that time China, Iran, Italy, Japan, and South Korea); or risk factors for infection complications (including age or co-morbid conditions); or the patient was a healthcare worker who could potentially expose others at risk. Test ordering was at the discretion of the attending emergency physician based on these criteria.

We used the ePLex SARS-CoV-2 test, which detects virus particles in clinical samples collected with a nasopharyngeal swab. The test was conducted under the GenMark Diagnostics platform with a US Food and Drug Administration emergency-use authorization, and evaluated in-house at our institution’s clinical laboratory. Data collected from patients who were tested included demographics, clinical characteristics, and test qualifying criteria. Demographics included age group (<18, 18–64, and 65+), gender, and race/ethnicity, Clinical characteristics included Emergency Severity Index (ESI) score, fever present on arrival to ED (yes/no), ED disposition, and COVID-19 test results. Test qualifying criteria included symptoms, contact with a person under investigation, a healthcare worker with potential contact of an infected person, recent travel to high-risk areas, and high-risk comorbidities. Patient demographics, clinical characteristics and test qualifying questions are presented overall and by test results with descriptive statistics.

## RESULTS

During the 10-day study period, the combined census of the study EDs was 2157 patient encounters. This was a decrease of about 21.2% from the same time period in 2019. A total of 283 tests were ordered in the ED. Patient demographics and clinical characteristics are presented in Table 1. The majority of patients were 18–64 years of age, male, non-Hispanic white, had an ESI score of three, did not have a fever, and were discharged from the ED. A total of 29 (10.2%) tested COVID-19 positive. Among these, characteristics paralleled the overall distribution of all patients that were tested. The majority (23/29, 79.3%) of COVID-19 positive patients were also discharged, left against medical advice, or eloped, while those who were admitted or transferred (6/29) were split between patients 18–64 years of age and 65 or older (three from each group). There have been no deaths in our cohort of COVID-19 patients.

The test qualifying criteria are reported in Table 2. Symptoms-based criteria most associated with COVID-19 were the most common criteria identified for testing (90.6%). All other criteria were reported in 43.0% or less of patients. Travel was the least common qualifying response (5.5%). We found only small differences in test qualifying criteria by symptoms and being a healthcare worker, between patients testing positive and negative. Having contact with a person under investigation was significantly more common in those testing positive vs negative (63% vs 24.5%, respectively). The majority of patients in both results groups had at least two qualifying criteria for testing (75.2% overall).

## DISCUSSION

COVID-19 infection is caused by a novel severe acute respiratory syndrome coronavirus 2 (SARS-CoV-2), a new human pathogen first identified in Wuhan, Hubei Province, China, in December 2019 that has led to worldwide pandemic. COVID-19 is similar to other zoonotic coronaviruses, named for the so-called “spike proteins” that appear like a crown on these enveloped viruses. A number of coronavirus types are “common cold” pathogens, while certain novel strains have led to outbreaks of respiratory diseases (SARS-CoV-1, Middle East respiratory syndrome (MERS-CoV).

Worldwide, as of March 27, 2020, there have been nearly 586,000 confirmed cases of COVID-19 resulting in 26,865 deaths. In the US there have been over 97,000 cases with over 1,400 deaths.^1^ For the majority of infections, COVID-19 results in a mild respiratory illness.^2^ However, a significant number result in serious morbidity and death, associated with advanced age and co-morbidities including hypertension, diabetes, and immunosuppression.^3^

The first case of COVID-19 confirmed in the US was reported in January 2020.^4^ The early stages of the response to COVID-19 in the US was hampered by multiple challenges and issues, particularly availability of diagnostic testing for the novel coronavirus.^5^ As a result, testing has been limited with specific criteria recommended by the CDC including severity of disease, such as requiring hospitalization, recent travel, or risk factors for significant morbidity and mortality.^6^ In response, a number of innovative approaches have been piloted to assess patients for the disease.^7^

As of mid-March, the CDC reported 7038 confirmed or presumptive positive coronavirus tests, with a total number of specimens tested by CDC labs of 4484 and US public health laboratories of 33,340.^8^,^9^ In our study, in the initial 10 days of in-house testing for ED patients meeting criteria for diagnostic evaluation, we report a 10.2% incidence rate of 283 tests conducted. The true incidence rate of COVID-19 in the US is unknown and will continue to be unclear until the ability for mass testing becomes available.

## LIMITATIONS

First, we report only preliminary data from the ED for an initial brief period (10 days). We believe this is one of the first reports of the results of in-house novel coronavirus testing in the emergency setting. Second, our study was conducted at a single healthcare institution with two EDs in one metropolitan region and thus our results may not reflect the conditions or expected findings in other communities or hospital EDs. Finally, this report is in the early stages of the pandemic in the US and in particular in the very early stages of testing availability in the US. It is likely that with further expansion of testing access and availability, new information and insights into this pandemic and its impact on our healthcare resources and communities will be discovered.

## CONCLUSION

In this review of prospectively collected data on all ED patients who had targeted testing for acute COVID-19 infection at two EDs in the first 10 days of testing, we found over 10% of those tested were identified as positive. Nearly all of these patients did not have a fever when they arrived to the ED. However, a history of viral infection symptoms was the most common criteria for testing. The continued monitoring of testing and results will help providers understand how COVID-19 is progressing in the community and identify patient characteristics most suggestive of acute infection. This will help with public health surveillance and ongoing efforts to reduce the transmission of the virus and “flatten the curve.”

## Figures and Tables

**Table 1:** Patient demographics and clinical characteristics by test results for patients who were tested for COVID-19 during the first 10 days of testing.

Characteristics	Positive (n = 29)		Negative (n = 254)		Total (n = 283)	
	Number	(%)	Number	(%)	Number	(%)
Age group						
<18	0	(0.0)	2	(0.8)	2	(0.7)
18–64	25	(86.2)	211	(83.1)	236	(83.4)
65+	4	(13.8)	41	(16.1)	45	(15.9)
Gender						
Female	13	(44.8)	120	(47.2)	133	(47.0)
Male	16	(55.2)	134	(52.8)	150	(53.0)
Race/ethnicity						
Hispanic	4	(13.8)	47	(18.5)	51	(18.0)
NH White	20	(69.0)	141	(55.5)	161	(56.9)
NH Black	0	(0.0)	13	(5.1)	13	(4.6)
NH Asian/PI	2	(6.9)	25	(9.8)	27	(9.5)
Other/Mixed/Unknown	3	(10.3)	28	(11.0)	31	(11.0)
Emergency severity index						
Resuscitation	0	(0.0)	1	(0.4)	1	(0.4)
Emergency	3	(10.3)	46	(18.3)	49	(17.5)
Urgent	14	(48.3)	120	(47.8)	134	(47.9)
Less urgent	11	(37.9)	72	(28.7)	83	(29.6)
Non-urgent	1	(3.4)	12	(4.8)	13	(4.6)
Fever present on arrival						
Yes	2	(6.9)	25	(9.9)	27	(9.6)
No	27	(93.1)	227	(90.1)	254	(90.4)
ED Disposition						
Admit/Transfer	6	(20.7)	75	(29.5)	81	(28.6)
Discharged/AMA/Eloped	23	(79.3)	179	(70.5)	202	(71.4)

Note: Missing measures included 2 temperature and 3 Emergency Severity Index values for patients with negative COVID-19 results.

*COVID-19*, coronavirus 2019; *NH*, non-Hispanic; *PI*, Pacific Islander; *ED*, emergency department; *AMA*, against medical advice.

**Table 2:** COVID-19 test qualifying question for patients who were tested for COVID-19 during the first 10 days of testing (n = 235/283 tested).

Qualifying questions	Positive (n = 27)		Negative (n = 208)		Total (n = 235)	
	Number	(%)	Number	(%)	Number	(%)
Symptoms						
Yes	25	(92.6)	188	(90.4)	213	(90.6)
No	2	(7.4)	20	(9.6)	22	(9.4)
Contact with person under investigation						
Yes	17	(63.0)	49	(23.6)	66	(28.1)
No	10	(37.0)	159	(76.4)	169	(71.9)
Healthcare worker						
Yes	7	(25.9)	51	(24.5)	58	(24.7)
No	20	(74.1)	157	(75.5)	177	(75.3)
Foreign travel to COVID endemic country						
Yes	3	(11.1)	10	(4.8)	13	(5.5)
No	24	(88.9)	198	(95.2)	222	(94.5)
Comorbidities						
Yes	8	(29.6)	93	(44.7)	101	(43.0)
No	19	(70.4)	115	(55.3)	134	(57.0)
Total number of confirmed qualifying questions						
0	0	(0.0)	3	(1.4)	3	(1.3)
1	4	(14.8)	51	(24.5)	55	(23.4)
2	15	(55.6)	123	(59.1)	138	(58.7)
3	7	(25.9)	30	(14.4)	37	(15.7)
4	0	(0.0)	1	(0.4)	1	(0.4)
5	1	(3.7)	0	(0.0)	235	(0.4)

Note: Questions were asked for 235 (83.0%) of the 283 patients who received COVID-19 testing.

*COVID-19*, coronavirus 2019.
